# Sensitivity of shotgun metagenomics to host DNA: abundance estimates depend on bioinformatic tools and contamination is the main issue

**DOI:** 10.1099/acmi.0.000104

**Published:** 2020-02-17

**Authors:** Andrew J. McArdle, Myrsini Kaforou

**Affiliations:** ^1^​ Section of Paediatric Infectious Disease, Department of Infectious Disease, Imperial College London, London W2 1PG, UK

**Keywords:** metagenomics, deep sequencing, taxonomy

## Abstract

A recent study reported that increasing host DNA abundance and reducing read depth impairs the sensitivity of detection of low-abundance micro-organisms by shotgun metagenomics. The authors used DNA from a synthetic bacterial community with abundances varying across several orders of magnitude and added varying proportions of host DNA. However, the use of a marker-gene-based abundance estimation tool (MetaPhlAn2) requires considerable depth to detect marker genes from low-abundance organisms. Here, we reanalyse the deposited data, and place the study in the broader context of low microbial biomass metagenomics. We opted for a fast and sensitive read binning tool (Kraken 2) with abundance estimates from Bracken. With this approach all organisms are detected even when the sample comprises 99 % host DNA and similarly accurate abundance estimates are provided (mean squared error 0.45 vs. 0.3 in the original study). We show that off-target genera, whether contaminants or misidentified reads, come to represent over 10 % of reads when the sample is 99 % host DNA and exceed counts of many target genera. Therefore, we applied Decontam, a contaminant detection tool, which was able to remove 61 % of off-target species and 79 % of off-target reads. We conclude that read binning tools can remain sensitive to low-abundance organisms even with high host DNA content, but even low levels of contamination pose a significant problem due to low microbial biomass. Analytical mitigations are available, such as Decontam, although steps to reduce contamination are critical.

## Data Summary

NCBI sequence read archive accession PRJNA521492

## Introduction

The study of metagenomics and microbiomes has yielded impressive insights into the microbiology of the environment and of multicellular organisms in health and disease [1].

Although more expensive than amplicon-based microbiome approaches (e.g. 16S rRNA gene sequencing), shotgun metagenomics is increasingly gaining prominence. Benefits include no PCR-related bias, greater specificity of identifications and representation of diversity, and ability to detect organisms from all kingdoms [2]. Additionally, metagenomic sequences can be analysed functionally, and whole or partial metagenomes can be reconstructed with greater depth of sequencing.

However, high-depth sequencing does not guarantee abundant microbial reads. Challenges most frequently arise when microbial biomass is low [3–5]. In this case, total DNA will be limited, and few reads may be obtained. Furthermore, the quantity of contaminant organisms is likely to remain constant (as the processes that cause contamination should not be not associated with the determinants of host DNA proportion), and thus their relative contribution will increase. The same problem can arise when samples are dominated by DNA from a host organism – in these cases, host sequencing reads may vastly outnumber those from microbes.

Although techniques exist to mitigate this by selectively depleting host DNA, usually by removing free DNA before lysis [6–9], they are in their infancy and could also deplete DNA from dead or damaged organisms, which would include those under immune attack [10]. Depleting host DNA would not reduce the impact of contamination occurring prior to depletion.

In this context, we commend Pereira-Marques *et al*. on their insightful study into the effects of host DNA and read depth on microbial abundance estimates from shotgun metagenomics [11].

The authors evaluated the impact of a range of amounts of host DNA and sequencing depths on microbiome taxonomic profiling using shotgun metagenomic sequencing, from synthetic samples where bacterial DNA from 20 species of varying abundances was spiked with varying amounts of murine DNA. Sequencing was performed to achieve 5.5 Gb per sample.

The authors showed that increasing proportions of host DNA (10, 90 and 99 %) led to decreased sensitivity in detecting very low- and low-abundance species, increasing the number of undetected species.

Although not stated, we anticipate the authors may have selected MetaPhlAn2 for their analysis because by detecting clade-specific marker genes of known number per organism, relative abundances within a sample can be directly estimated [12]. Despite this advantage, we are concerned that relying upon a small number of marker genes will render the approach less resilient to the pitfalls of reduced depth than read binning approaches.

Consequently, we applied Kraken, a fast and sensitive read binning tool [13], which performed well in recent benchmarks [14, 15]. Advantageously, a partner tool (Bracken) also exists for relative abundance estimation [16]. We obtained the variable-length trimmed reads from the study (NCBI sequence read archive accession PRJNA521492) and built a Kraken database comprising NCBI RefSeq bacterial, fungal, viral, archaeal and mouse genome sequences with core vector elements (downloaded on 5 July 2019 using included scripts). This resulted in 18 834 operational taxonomic units (OTUs). Kraken (version 2.0.8-beta) was then run with default settings, followed by Bracken.

For each sample we categorized reads assigned to any microbial OTU as microbial. We follow the sample naming conventions of the original analysis: MS=microbial sample; SS10=10 % host DNA; SS90=90 % host DNA; SS99=99 % host DNA.

## Sensitivity

All expected organisms (*n*=20) were detected in all samples. This contrasts with the results presented by Pereira-Marquez *et al*. where nine of the 20 species became undetectable in SS99.

Over 75 % of microbial reads were allocated to the known species (on target), except in sample SS99 where this fell to 67 %. Other species of the expected genera represented much fewer than 1 % of microbial reads in all samples. Fewer than 2 % of microbial reads were assigned to OTUs outside of the lineage of the expected genera (off target), except for SS99 where this was 12 % (Table S1, available in the online version of this article).

## Relative abundance

Crude assigned read counts are not a guide to relative abundance because of varying genome size, and because reads from different organisms may be assigned at the species level at differing rates due to homology. Bracken was developed to overcome the second limitation by reallocating reads assigned to higher levels. We apply Bracken here at the species level to estimate abundance and then correct for genome size. The Bracken database was built for a read length of 150 (the median length of the trimmed reads).

Bracken estimated that over 98 % of microbial reads were on-target (species) in MS and SS10. In SS90 this fell to 96.8 % and in SS99 to 83.3 %.

**Fig. 1.**
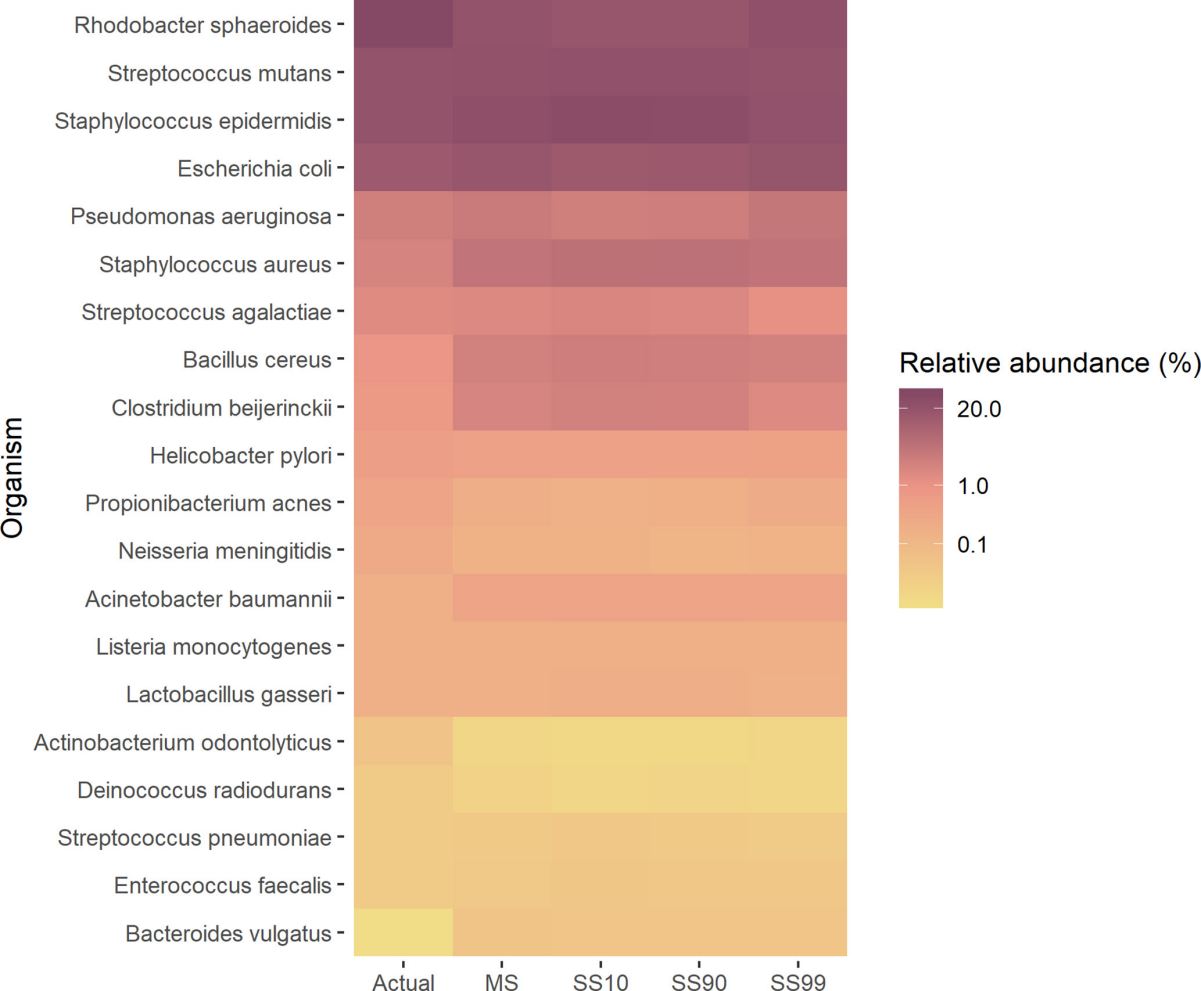
Taxonomic profile of the synthetic metagenome samples determined with Kraken 2, and expressed as the relative abundance of species in a heat map. Actual abundances are presented as per the original publication based on the theoretical number of genome copies present. Species are listed from highest to lowest expected relative abundances. MS=microbial sample; SS10=10 % host DNA; SS90=90 % host DNA; SS99=99 % host DNA.

We normalized abundances by genome size (obtained from NCBI genomes at https://www.ncbi.nlm.nih.gov/genome) for the target species, discounting the small proportion of off-target reads. In MS, the ratios of observed/expected relative abundance was between 0.5 and 2 for 16 of the 20 species, compared to 17 in the published study (Fig. 1 and Table S2). The mean squared relative error for MetaPhlAn was 0.3 and for Bracken was 0.45.

Changes in relative abundance due to host DNA abundance were modest, even in SS99 where 12 of 20 organisms were within 10 % of the estimate from MS (mean squared relative error 0.02; Table S2).

We found the association of variation in observed/expected ratio with genome GC content to be similar to the original report (*r*=−0.74 vs. −0.85; data not shown).

## Other species

Using Bracken recalculated reads, off-target genera (*n*=1 336) could be classified into synthetic-associated (MS:SS99 >10 : 1), host-associated (SS99:MS >10 : 1) or non-specific. Over 92 % of reads were from host- or synthetic-associated genera. Synthetic-associated genera contributed 0.8 % of microbial reads in MS, and host-associated genera less than 0.01 %. Host-associated genera contributed 11.5 % of microbial reads in SS99 (despite being only 0.2 % of murine reads), and synthetic-associated genera 1 %.

The top four synthetic-associated genera were *Shigella *, *Salmonella *, *Citrobacter* and *Klebsiella *. These are all likely to represent misclassified *Escherichia coli* reads. The top four host-associated genera were *Pasteurella *, *Halomonas *, *Alcanivorax* and *Mycobacteria. Alcanivorax* and *Pasteurellaceae* have previously been reported to contaminate DNA extraction kits [17]. We note that host DNA was extracted in the laboratory whereas the microbial DNA was obtained commercially, and thus different contaminants are unsurprising.

The target genera with lowest read counts in SS99 were *Schaalia* and *Deinococcus* (36 and 37 reads respectively). Fifty-four off-target genera had 36 or more reads. The most abundant off-target genus (* Pasteurella *) contributed 11 530 reads, greater than 13 of 17 target genera.

## Low microbial biomass

The greater sensitivity of this read binning approach reveals the underlying problem of high relative contamination in the samples with high host DNA content. The problem can now be reframed as one of low (proportionate) microbial biomass and potential mitigations can be considered.

The challenge of low microbial biomass samples, introduced earlier, has been more extensively studied in rRNA amplification-based approaches than shotgun metagenomics. Nonetheless, many of the problems are shared, and we direct readers to a recent review by Eisenhofer *et al*. [3]. Pre-analytical mitigations include appropriate controls, as described therein.

Analytical mitigations for 16S rRNA gene studies were explored in a recent publication [5]. The authors investigated filtering based on relative abundance thresholds in negative controls: Decontam [18], an approach based on the inverse relationship between the relative abundance of contaminants and total microbial DNA; and SourceTracker [19], which takes a Bayesian approach using external or internal community references.

In summary, it was found that simple censoring of thresholded negative control OTUs discriminated contaminant and target sequence variants poorly. The Decontam approach discriminated better, correctly classifying all target sequence clusters, and up to 90.4 % of contaminant sequence clusters. SourceTracker performed poorly without external references (a typical scenario), identifying less than 1 % of contaminant sequence clusters.

Although limited by few samples and no duplicates, we applied Decontam to the Bracken-normalized species counts, using the frequency-based approach. Input DNA concentration was replaced by the total microbial read counts (because all samples had been normalized to 0.2 ng ml^−1^). None of the 20 target species were classified as contaminants. In total, 2636 of 4319 (61 %) off-target species were classified as contaminants, and these accounted for 92 % of off-target reads in SS99 and 68 % in SS90. Only 11 % of off-target reads in SS10 and 5 % in MS were classified as contaminants, unsurprisingly, as these reads are dominated by synthetic-associated genera.

In SS99, the least abundant genera, *Schaalia* and *Deinococcus *, retained 35 and 34 reads, respectively. Only seven off-target genera had 34 or more reads, comprising the four synthetic-associated genera above, with *Cronobacter *, *Nitrosopumilus* and *Enterobacter. Shigella* had the most reads at 1303, exceeding 10 of 17 target genera.

## Interpretation

The marker gene approach employed by MetaPhlAn is very sensitive to read depth, and hence to host DNA abundance. In contrast, the read binning approach employed by Kraken 2 detects organisms across the >2 000-fold range of relative abundances even with 99 % host DNA content.

Genome-size normalization of Bracken-estimated read counts provides similarly accurate estimates of relative abundance to MetaPhlAn. The untrimmed reads (not available) may give better results as they would all be of the same length, which is expected by Bracken.

We demonstrate that the large relative contribution of contaminants when microbial reads are in a minority is a greater concern, representing around 10 % of microbial reads in SS99 with contaminant genera exceeding the counts of some target genera.

However, the frequency-based Decontam approach allows nearly four-fifths of these off-target reads to be excluded. Furthermore, many of those that remain may represent misclassified target reads.

It is important to note that the literature does not demonstrate supremacy of read binning approaches in all regards. Walsh *et al*. [20] showed in a low-complexity food microbiome that Metaphlan 2 was sensitive and also more specific than Kraken.

## Concluding remarks

The appropriate selection of analytical tools is vital for accurate and sensitive metagenome analysis. For samples with low microbial biomass, reducing contamination is a priority, although mitigation is possible. Techniques to selectively remove host DNA are required, but thorough benchmarking is awaited.

## Data bibliography

Code and output from Kraken and Bracken are available at https://github.com/andrewjmc/pereira_marques_reanalysis

## Supplementary Data

Supplementary material 1
